# Will measuring mechanical properties help us understand solid-state reactions?

**DOI:** 10.1107/S2052252515019922

**Published:** 2015-10-30

**Authors:** Ian D. Williams

**Affiliations:** a Department of Chemistry, Hong Kong University of Science and Technology, Clear Water Bay, Hong Kong

**Keywords:** photomechanical properties, solid-state reactions, crystal engineering, cinnamic acid, nanoindentation

## Abstract

In the solid-state photodimerization of cinnamic acid polymorphs, can mechanical properties tell us whether Schmidt ‘minimal molecular movement’ or Kaupp ‘molecular migration’ is more important?

The study of solid-state reactions is clearly an important area where crystallography can directly contribute to the understanding of what is occurring. It is also an area of science where two apparently contradictory viewpoints surfaced to explain such phenomena. Mishra *et al.* (2015 ▸) looks at the photodimerization reaction of two polymorphs of a cinnamic acid derivative. These have different photomechanical properties that may indicate that in one Schmidt’s ‘minimal molecular movement’, and in the other Kaupp’s ‘molecular migrations’ can dominate in these photoreactions. We look back at how two seemingly different ideas were reached and how their successful reconciliation gives us a deeper understanding of the issues involved.

In 1964, Gerhard Schmidt published three celebrated articles back-to-back in the *Journal of the Chemical Society* on topochemical 2+2 photodimerizations of a variety of *trans*-cinnamic acids (Schmidt, 1964 ▸; Cohen & Schmidt, 1964 ▸; Cohen *et al.*, 1964 ▸). Certain ‘topochemical principles’ for such solid-state photoreactions – the orientation, alignment and spacing of reactive groups – were gleaned from these studies and one criterion was that the midpoints of the reactive double bonds should be within a threshold distance, *d*, of around 4.0 Å (Schmidt, 1971 ▸). Although topochemical (a term first used by Kohlschütter, 1919 ▸), the reactions were rarely topotactic, or single-crystal-to-single-crystal, in nature. However, this was shown to be possible by using ‘tail absorption’ by Enkelmann *et al.* (1993 ▸) who beautifully demonstrated the progressive structural change of α-*trans*-cinnamic acid itself to the head-to-tail α-truxillic acid dimer product. This proceeded with ‘minimal atomic motion’ in the crystal, another axiom of Schmidt and could also be taken to effectively quantitative 100% conversion. This groundwork of Schmidt’s helped pave the way for the highly active and broader field we define as ‘crystal engineering’, a term he coined in 1971 (Schmidt, 1971 ▸), shortly before his death. However, as with all scientific hypotheses, the criteria for the 2+2 reactions were also open to verification and revision.

Over time a growing number of examples were found to violate these principles. For example, some phases were unreactive despite the proximity of reactive groups, whilst others underwent reaction although their reactive groups lay well beyond the threshold limit. In the 1980s, the groups of Gerd Kaupp and Fumio Toda rekindled interest in organic solid-state synthesis *via* gas–solid and solid–solid reactions facilitated by grinding (Kaupp & Mathies, 1986 ▸; Toda, 1987 ▸). They also explored the mechanisms behind a wide range of such ‘solvent free’ reactions, which could frequently proceed to 100% completion (Kaupp *et al.*, 2001 ▸). Kaupp developed a three-step solid-state reaction theory (Kaupp, 2003 ▸), backed by evidence from atomic force microscopic (AFM) data. This involved phase rebuilding, phase transformation and crystal disintegration steps. Crucially, and in opposition to the core of Schmidt’s idea, this was more favorable where long-range atomic or molecular movements were feasible. If they were not, then reactions would slow to a halt and not reach completion. Kaupp highlighted several violations of ‘Schmidt topochemistry’ in his review of solid-state molecular syntheses that he felt could be explained by the three-step model (Kaupp, 2003 ▸).

However, in these 2+2 photoreactions the ideas of *both* Schmidt *and* Kaupp can make valuable contributions that are not necessarily exclusive. For any chemical reaction to occur the transition state must be attained. For these photodimerizations the reactive alkenes, including one molecule already in a photo-excited state, *must* be sufficiently proximal for π–π overlap leading to formation of the cyclobutane ring. This is the origin of Schmidt’s criterion of centroid proximity from a static crystal structure. Through molecular motion in a dynamic crystal this could also be achieved through a Kaupp-type process. Another aspect of the formation of the ring-like transition state is not just a closure of the alkene C—C separations, but also the necessary rehybridization of their C atoms from planar *sp* ^2^ towards tetrahedral *sp* ^3^. This requires considerable atomic motion for the aryl and carboxylate groups. Hence, in brittle, inelastic crystals this is disfavored, possibly resulting in lower reaction rates, or in the extreme perhaps even becoming a ‘Schmidt violation’. The concept of a flexible space to assist reaction rate can be traced to Cohen (1975 ▸) and his idea of a ‘reaction cavity’. Ironically, the presence of bulkier aryl groups and larger separations between C=C bonds can actually give more freedom to the needed rearrangement.

The study of the two polymorphs of 3,4-dimethoxycinnamic acid (DCMA) present some interesting contrasts (Mishra *et al.*, 2015 ▸). The original triclinic form I (Desiraju *et al.*, 1984 ▸) has a layer arrangement with obvious slip or cleavage planes. Although only half of the molecules are set up for photoreaction, it is observed that the crystals exhibit photosalient behavior and jump after irradiation. This is consistent with ‘Kaupp-behavior’ and release of strain through massive dislocations in the slip planes. By contrast, in the newly discovered form II the molecular packing has an interlocking zigzag pattern. The molecular conformations are more similar to the final product truxillic acid and so the photoreaction in this polymorph is more ‘Schmidt-like’.

The use of a nanoindentation method confirms the higher mechanical stiffness of the new form II. Whether this technique will be a game changer in understanding the many complexities of solid-state reactions remains to be seen. The nanoindentation method requires access to suitable crystal faces, which might be limited in some organic crystals. Overall it is clear that measurement of mechanical properties of organic crystals has long been neglected and a ready technique that can be applied at the level of relatively small individual crystallites could provide useful insights not otherwise available.

Bringing ancillary techniques to bear on a problem can give surprises, as well. For example through ^13^C-CPMAS solid-state NMR studies, Fonseca *et al.* (2009 ▸) have shown that reaction rate is not well correlated with C=C separation and reaction in α-*o*-ethoxycinnamic acid (*d* = 4.53 Å) is 2× that of the *o*-methoxy analogue (*d* = 4.12 Å). It appears to propagate in a two-dimensional manner from internal nuclei, rather than in a one-dimensional manner from the surface. Careful ^13^C-CPMAS NMR studies of the photodimerization of the two DCMA polymorphs and conversely nanoindentation measurements on systems such as the α-forms of *o*-methoxy- and *o*-ethoxycinnamic acids would provide a useful cross-correlation study of the two methods and indicate whether mechanical properties can indeed dominate in the progress of these solid-state reactions.
